# 抑制Src酪氨酸激酶对非小细胞肺癌细胞分泌MMP-2和MMP-9的影响

**DOI:** 10.3779/j.issn.1009-3419.2011.01.03

**Published:** 2011-01-20

**Authors:** 锐 郑, 晓松 秦, 文洁 李, 健 康

**Affiliations:** 1 110022 沈阳，中国医科大学附属盛京医院第二呼吸内科 2^nd^ Department of Respiratory Medicine, Shengjing Hospital of China Medical University, Shenyang 110022, China; 2 110004 沈阳，中国医科大学附属盛京医院检验科 Department of Clinical Laboratory, Shengjing Hospital of China Medical University, Shenyang 110004, China; 3 110001 沈阳，中国医科大学附属第一医院呼吸疾病研究所 Institute of Respiratory Disease, the First Hospital Afiated to China Medical University, Shenyang 110001, China

**Keywords:** Src酪氨酸激酶, 肺肿瘤, MMP-2, MMP-9, 浸润, Src tyrosine kinase, Lung neoplasms, MMP-2, MMP-9, Invasion

## Abstract

**背景与目的:**

Src酪氨酸激酶和基质金属蛋白酶在肺癌的浸润和转移中发挥重要作用。本研究旨在探讨抑制Src酪氨酸激酶对非小细胞肺癌（non-small cell lung cancer, NSCLC）细胞分泌基质金属蛋白酶-2（matrix metalloproteinase 2, MMP-2）和基质金属蛋白酶-9（matrix metalloproteinase 9, MMP-9）以及NSCLC细胞侵袭浸润的影响。

**方法:**

采用ELISA法检测NSCLC细胞（PC14PE6、H226、PC-9、A549）培养上清中MMP-2和MMP-9含量以及抑制Src酪氨酸激酶对NSCLC细胞分泌MMP-2和MMP-9的影响；Boyden chamber法检测抑制Src酪氨酸激酶对NSCLC细胞体外侵袭浸润的影响。

**结果:**

NSCLC细胞中PC14PE6和H226中MMP-2和MMP-9的水平较高，A549细胞中MMP-9的水平较低，而MMP-2和MMP-9在PC-9细胞中检测不到。Src酪氨酸激酶抑制剂对PC14PE6中的MMP-2水平以及PC14PE6、H226和A549细胞中的MMP-9水平呈剂量依赖性抑制关系。10 µM Src酪氨酸激酶抑制剂使PC14PE6细胞中的MMP-2水平、H226细胞和A549细胞中的MMP-9水平降低50%以上。10 µM Src酪氨酸激酶抑制剂对H226细胞中的MMP-2无明显抑制作用。Src酪氨酸激酶抑制剂对4种NSCLC细胞体外侵袭浸润的抑制程度略有差异，但均呈现明显的剂量依赖性抑制作用。3 µM Src酪氨酸激酶抑制剂对PC14PE6、H226、A549和PC-9细胞体外侵袭浸润的抑制率分别为79.1%、68.09%、90.96%和96.98%（*P* < 0.001）。

**结论:**

通过抑制NSCLC细胞分泌MMP-2和MMP-9，抑制Src酪氨酸激酶可降低细胞的体外侵袭浸润能力。

## Abstract (en)

**Background and objective:**

Src tyrosine kinase and matrix metalloproteinase play the pivotal roles in lung cancer invasion and metastasis. The aim of this study is to evaluate the effect of Src tyrosine kinase inhibition on secretion of matrix metalloproteinase 2 (MMP-2) and matrix metalloproteinase 9 (MMP-9) by non-small cell lung cancer (NSCLC) cells.

**Methods:**

ELISA was used to examine the activity of MMP-2 and MMP-9 produced by NSCLC cells (PC14PE6, H226, PC-9, A549) as well as the effect of Src tyrosine kinase inhibition on secretion of MMP-2 and MMP-9 by NSCLC cells. Boyden chamber assay was used to assess the effect of Src tyrosine kinase inhibition on invasion of NSCLC cells *in vitro*.

**Results:**

The levels of MMP-2 and MMP-9 in PC14PE6 and H226 cells were high, whereas the level of MMP-9 in A549 cell was low. MMP-2 and MMP-9 levels in PC-9 cell could not be detected. Src tyrosine kinase inhibitor obviously decreased the secretion of MMP-9 by PC14PE6, H226 and A549 cells, as well as MMP-2 by PC14PE6 cells in a dose-dependent manner. 10 µM Src tyrosine kinase inhibitor suppressed the secretion of MMP-9 by H226 and A549 cells, as wells as MMP-2 by PC14PE6 cells by more than 50%, while the same concentration of Src tyrosine kinase inhibitor almost had no effect on the level of MMP-2 in H226 cell. Invasiveness of NSCLC cells was suppressed by Src tyrosine kinase inhibitor in a dose-dependent manner, though there was minor difference in degree of the inhibition among four cell lines. 3 µM Src tyrosine kinase inhibitor suppressed the cell invasiveness of PC14PE6, H226, A549 and PC-9 cells by 79.1%, 68.09%, 90.96% and 96.98%, respectively (*P* < 0.001).

**Conclusion:**

Inhibition of Src tyrosine kinase could suppress the invasion of NSCLC cells as well as the secretion of MMP-2 and MMP-9 by NSCLC cells *in vitro*. MMP-2 and MMP-9 were involved in regulating cell migration and invasion.

基质金属蛋白酶（matrix metalloproteinase, MMP）是一组金属离子依赖的蛋白酶，主要分为胶原酶、明胶酶、基质溶解素和膜型MMPs（MT-MMPs）4种类型^[1]^。细胞间质中的成纤维细胞、内皮细胞、平滑肌细胞、巨噬细胞和中性粒细胞等均可分泌MMPs。它们能降解多种细胞外基质成分，包括胶原、层联蛋白、纤维联结蛋白、弹性蛋白和蛋白聚糖等^[2]^。MMPs与肿瘤细胞浸润突破基底膜、穿透血管、原位和远处转移以及转移瘤的生长和血管形成密切相关^[3]^。包括肺癌在内的人类很多肿瘤均存在MMPs特别是明胶酶A（MMP-2）和明胶酶B（MMP-9）、MT1-MMP以及基质溶解素-3（MMP-11）的过度表达^[4, 5]^。

原癌基因*C-Src*是生长因子受体信号通路中的重要成分，在调控细胞的生长、粘附、运动和细胞信号转导等方面发挥重要作用，其异常表达和活化与肿瘤的发生、发展和转移密切相关^[6]^。研究^[7, 8]^表明，抑制Src酪氨酸激酶活性能够降低胰腺癌和前列腺癌细胞中MMP-2和MMP-9的活性，抑制癌细胞的侵袭和浸润。本研究应用选择性Src酪氨酸激酶抑制剂作用于NSCLC细胞株，旨在研究抑制Src酪氨酸激酶对非小细胞肺癌（non-small cell lung cancer, NSCLC）细胞分泌MMP-2和MMP-9的影响以及对NSCLC细胞体外侵袭浸润的影响。

## 1 材料与方法

### 1.1 主要试剂、药品和设备

人肺腺癌PC14PE6细胞和鳞癌H226细胞为美国德州大学安德森癌症中心所赠；人肺腺癌A549细胞购于美国模式培养物保藏所；人肺腺癌PC-9细胞购于日本IBL公司。选择性Src酪氨酸激酶抑制剂4-苯胺喹唑啉衍生物由英国AstraZeneca公司提供。Biotrak MMP-2和MMP-9活性分析试剂盒购自英国Amersham Pharmacia Biotech公司。胶原Ⅰ购自BD Biosciences公司。纤维联结蛋白购于日本岩城硝子公司。Transwell chambers购自Costar Cambridge。Diff-Quik染色系统购自美国Baxter Scientifc Products McGraw Park IL。

### 1.2 细胞培养

PC14PE6细胞在含10 %小牛血清的RPMI-1640培养基中，A549细胞、PC-9细胞和H226细胞在含10%小牛血清的DMEM培养基中，于37 ℃、5%CO_2_饱和湿度培养。每毫升培养液含100 U青霉素、100 µg链霉素。

### 1.3 ELISA法检测MMP-2和MMP-9含量^[9]^

NSCLC细胞4×10^5^/mL接种于6孔板，于无血清的DMEM培养液中孵育24 h，用不同浓度的Src酪氨酸激酶抑制剂（0.1 µM、1 µM、3 µM和10 µM）37 ℃下孵育6 h，收集培养上清，用ELISA法检测NSCLC细胞培养上清中MMP-2和MMP-9的含量。MMP-2和MMP-9的检出限分别为0.75 ng/mL和0.25 ng/mL。

### 1.4 改良的Boyden chamber方法检测Src酪氨酸激酶抑制剂对肺癌细胞侵袭浸润的抑制作用^[10]^

使用胶原Ⅰ（30 μg/filter）包埋孔径为8 μm的transwell小室。血清饥饿24 h的肺癌细胞（1×10^5^/200 μL）悬浮于含有不同浓度Src酪氨酸激酶抑制剂（0.1 µM、0.3 µM、1 µM和3 µM）的培养液中，加到槽的上层。含有10 μg/mL纤维联结蛋白的趋化液加到槽的下层。置37 ℃、5%CO_2_培养箱中孵育6 h后，用棉棒擦掉未浸润到下层的细胞，切下分隔膜，使用Diff-Quik系统进行固定和染色。在200倍亮视野显微镜下随机选取6个视野计数细胞数，取3个独立实验的平均值进行统计学分析。在进行侵袭浸润实验的同时，用MTT法检测Src酪氨酸激酶抑制剂对细胞生存活力的影响。

### 1.5 统计学分析

采用SPSS 11.0统计分析软件进行方差分析，*P* < 0.05为差异有统计学意义。

## 2 结果

### 2.1 NSCLC细胞株中MMP-2和MMP-9的表达水平

ELISA结果显示，在4株NSCLC细胞系中，MMP-2和MMP-9均可在PC14PE6和H226细胞中表达，两者培养上清中MMP-2水平分别为9.21 ng/mL和2.28 ng/mL，MMP-9水平分别为0.87 ng/mL和0.75 ng/mL。A549细胞培养上清中MMP-9水平为0.57 ng/mL，A549细胞中MMP-2水平以及PC-9细胞中的MMP-2和MMP-9水平均检测不到（图 1）。

**1:**
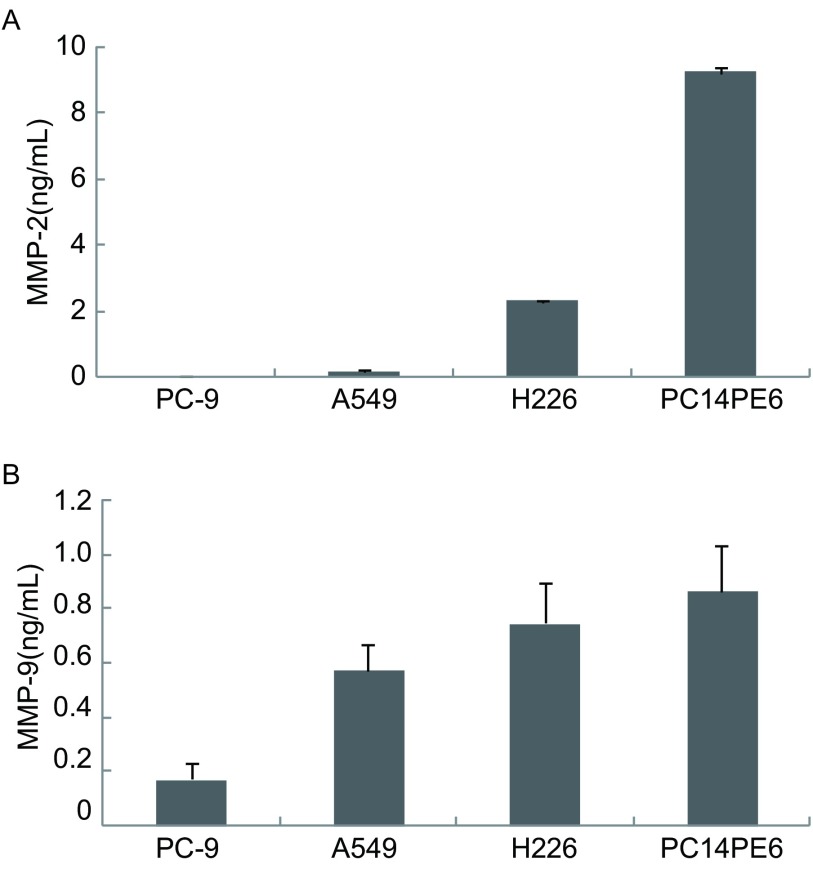
NSCLC细胞株中MMP-2、MMP-9的分泌情况。A：MMP-2；B：MMP-9。 Secretion of MMP-2, MMP-9 by NSCLC cell lines. A: MMP-2; B: MMP-9.

### 2.2 Src酪氨酸激酶抑制剂对PC14PE6和H226细胞中MMP-2水平的影响

如图 2所示，Src酪氨酸激酶抑制剂对PC14PE6细胞中MMP-2的水平呈剂量依赖性抑制关系，当浓度达到10 µM时，其对PC14PE6中MMP-2的抑制程度超过50%。相同浓度的Src酪氨酸激酶抑制剂对H226细胞中的MMP-2水平无明显抑制作用。

**2:**
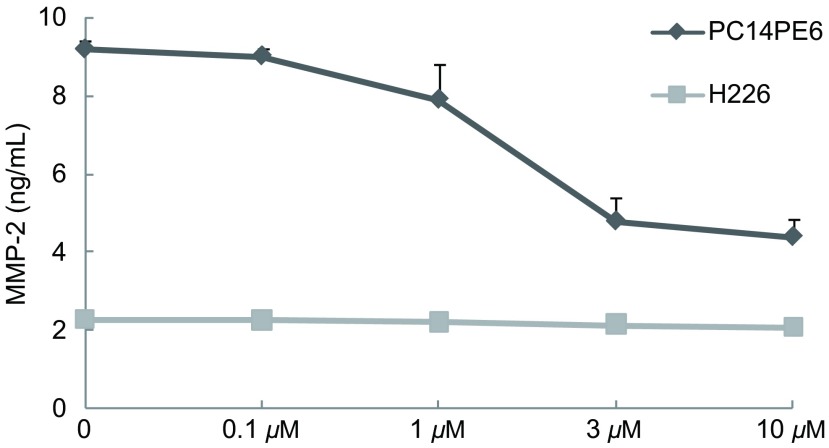
Src酪氨酸激酶抑制剂对PC14PE6和H226细胞分泌MMP-2的影响 Effect of Src tyrosine kinase inhibitor on secretion of MMP-2 by PC14PE6 and H226 cells

### 2.3 Src酪氨酸激酶抑制剂对PC14PE6、H226和A549细胞中MMP-9水平的影响

如图 3所示，Src酪氨酸激酶抑制剂与PC14PE6、H226和A549细胞中的MMP-9呈剂量依赖性抑制关系。10 µM Src酪氨酸激酶抑制剂使H226细胞和A549细胞中的MMP-9水平降低50%以上。

**3:**
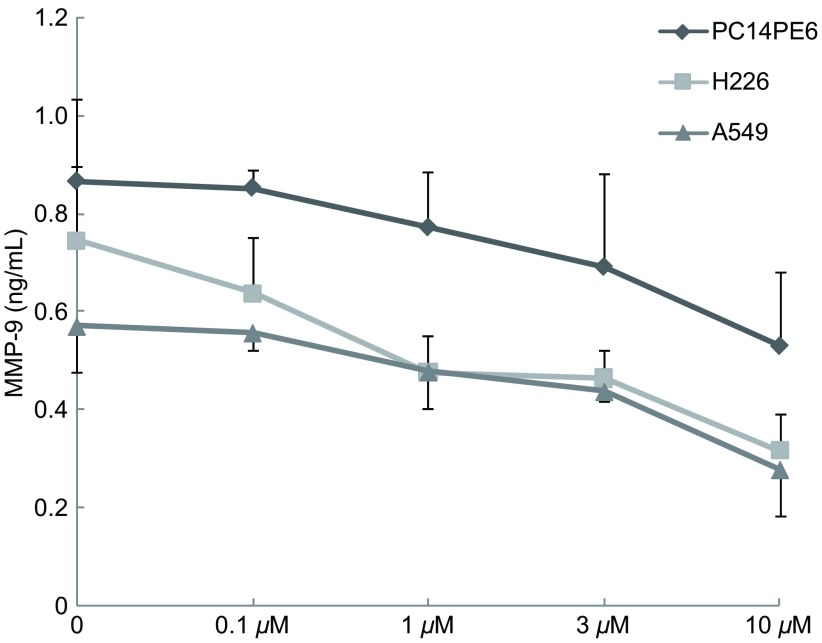
Src酪氨酸激酶抑制剂对PC14PE6、H226和A549细胞分泌MMP-9的影响 Effect of Src tyrosine kinase inhibitor on secretion of MMP-9 by PC14PE6, H226 and A549 cells

**4:**
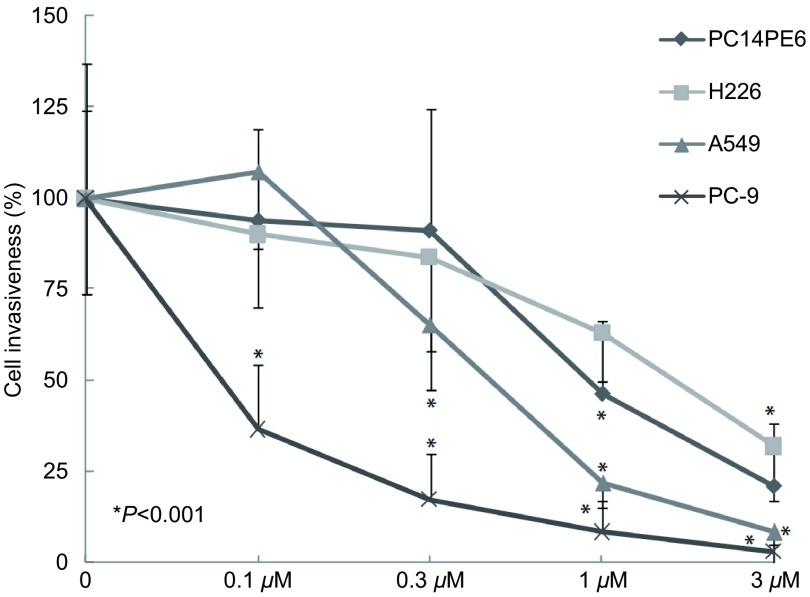
Src酪氨酸激酶抑制剂对NSCLC细胞体外侵袭浸润的影响 Effect of Src tyrosine kinase inhibitor on NSCLC cell invasiveness

### 2.4 Src酪氨酸激酶抑制剂对4种NSCLC细胞体外侵袭浸润的影响

Src酪氨酸激酶抑制剂对NSCLC细胞体外侵袭浸润具有明显的抑制作用，对不同细胞的抑制程度略有差别。1 μM和3 μM Src酪氨酸激酶抑制剂对分泌高水平MMP-2和MMP-9的PC14PE6和H226细胞体外侵袭浸润具有明显的抑制作用，抑制率分别为54.73%（*P* < 0.001）和79.1%（*P* < 0.001）以及36.88%（*P* < 0.001）和68.09%（*P* < 0.001）。0.3 μM、1 μM和3 μM Src酪氨酸激酶抑制剂对仅分泌MMP-9的A549细胞体外侵袭浸润作用更敏感，抑制率分别为36.35%（*P* < 0.001）、77.9%（*P* < 0.001）和90.96%（*P* < 0.001）。而0.1 μM、0.3 μM、1 μM和3 μM Src酪氨酸激酶抑制剂对检测不出MMP-2和MMP-9的PC-9细胞体外侵袭浸润的作用最敏感，抑制率分别为63.46%（*P* < 0.001）、82.69%（*P* < 0.001）、91.67%（*P* < 0.001）和96.98%（*P* < 0.001）。

## 3 讨论

上皮肿瘤转移涉及一系列的复杂过程，而细胞浸润突破基底膜被认为是最关键的步骤^[6]^。由于MMPs能降解基底膜和细胞外基质中的各种成分，所以它在此过程中发挥重要作用。MMPs的过度表达和活化与肿瘤生长、侵袭和转移密切相关^[1, 3, 5, 11]^。肿瘤细胞可以通过可溶性递质或膜结合分子与间质细胞进行信息交换，协同产生和调节MMPs，这在肿瘤细胞侵袭和转移的机制中具有重要意义^[5]^。MMPs过度表达在包括肺癌在内的许多恶性肿瘤中均与预后不良有关^[12-14]^。目前已鉴定出30多种结构有关联的MMPs，其中分子量为72 kDa和92 kDa的MMP-2和MMP-9能降解多种胶原、明胶、弹性蛋白和层联蛋白等，在人类很多肿瘤中都有很高的表达和活化，是肺癌中主要的蛋白水解酶^[4, 5]^。肺癌组织中MMP-2及MMP-9表达水平的上调使肺癌细胞具有降解基底膜及细胞外基质的能力，肺癌细胞突破各种屏障的能力增强，侵袭转移能力增强^[15]^。

本研究发现，NSCLC细胞中PC14PE6和H226细胞株表达高水平的MMP-2和MMP-9，A549细胞株表达低水平的MMP-9，而MMP-2和MMP-9在PC-9细胞株中低于检测限。结合前期的研究^[9]^，PC14PE6和H226细胞中明胶酶活性可能归于MMP-2和MMP-9，A549细胞明胶酶活性可能主要归于其它的蛋白水解酶，如胰蛋白酶，部分活性归于MMP-9。在肿瘤细胞浸润和转移过程中MMPs的表达是多水平严格调控的，包括基因的活化、转录、翻译、蛋白酶的分泌、酶原的激活、特异性抑制因子以及活性酶的降解和清除。其中关键的环节是转录水平和酶原的激活。细胞内MAPK系统亦参与MMPs的表达调控，其途径为多种生长因子、细胞因子通过其膜受体激活Ras/Raf/MEK/MAPK（ERK1/2或p38）通路，通过转录因子AP-1和EST调节MMPs的表达^[16]^。

Src蛋白能和许多生长因子受体相互作用，在细胞信号转导、MMPs活性以及细胞侵袭浸润的调节中发挥重要作用。研究^[7, 8]^表明，Src酪氨酸激酶抑制剂（> 2 μM）能够抑制前列腺癌PC3细胞中MMP-9的活性以及胰腺癌细胞中MMP-2和MMP-9的活性。本研究发现，高浓度（≥3 μM）Src酪氨酸激酶抑制剂可明显抑制NSCLC细胞PC14PE6、H226和A549细胞分泌MMP-9以及PC14PE6细胞分泌MMP-2。而在细胞侵袭浸润实验中，亚微摩尔水平Src酪氨酸激酶抑制剂明显抑制PC-9细胞的体外侵袭浸润，1 μM Src酪氨酸激酶抑制剂能明显抑制A549、PC14PE6和H226细胞的体外游走浸润。由此可见，抑制NSCLC细胞游走浸润所需要的Src酪氨酸激酶抑制剂的浓度明显低于抑制MMPs活性所需要的药物浓度。由于PC-9细胞中MMP-2和MMP-9活性检测不出，推测Src酪氨酸激酶不是通过调节MMPs活性来促进PC-9细胞的游走浸润。而在NSCLC细胞A549、PC14PE6和H226中，Src酪氨酸激酶对细胞侵袭浸润的调节不仅是通过调节MMP-2和MMP-9的活性来发挥作用的。Src酪氨酸激酶还可以通过p190RhoGAP和粘着斑激酶的磷酸化调控细胞骨架的重组，调节细胞粘附和运动^[6, 17]^，促进上皮基质转化，从而削弱正常上皮间的粘附，刺激肿瘤细胞的侵袭和浸润。

综上所述，NSCLC细胞分泌MMP-2和MMP-9呈现细胞异质性，抑制Src酪氨酸激酶能够抑制NSCLC细胞分泌MMP-2和MMP-9以及NSCLC细胞的体外侵袭浸润。MMP-2和MMP-9参与Src酪氨酸激酶介导的某些NSCLC细胞体外侵袭浸润，但不是唯一决定因素。
